# Small-molecule AgrA inhibitors F12 and F19 act as antivirulence agents against Gram-positive pathogens

**DOI:** 10.1038/s41598-018-32829-w

**Published:** 2018-10-01

**Authors:** Michael Greenberg, David Kuo, Eckhard Jankowsky, Lisa Long, Chris Hager, Kiran Bandi, Danyang Ma, Divya Manoharan, Yaron Shoham, William Harte, Mahmoud A. Ghannoum, Menachem Shoham

**Affiliations:** 1 Department of Biochemistrty, Case Western Reserve University School of Medicine, Cleveland, OH 44106 USA; 2 Center for RNA and Therapeutics, Case Western Reserve University, Cleveland, OH 44106 USA; 3 Department of Dermatology, Case Western Reserve University School of Medicine, Cleveland, OH 44106 USA; 4 Present Address: University of Pittsburgh School of Medicine, Pittsburgh, USA; 5 Q2 Pharma, Ltd., Haifa, Israel; 6 Case Western Reserve University, Cleveland, OH 44106 USA

## Abstract

Small-molecule antivirulence agents represent a promising alternative or adjuvant to antibiotics. These compounds disarm pathogens of disease-causing toxins without killing them, thereby diminishing survival pressure to develop resistance. Here we show that the small-molecule antivirulence agents F12 and F19 block staphylococcal transcription factor AgrA from binding to its promoter. Consequently, toxin expression is inhibited, thus preventing host cell damage by Gram-positive pathogens. Broad spectrum efficacy against Gram-positive pathogens is due to the existence of AgrA homologs in many Gram-positive bacteria. F12 is more efficacious *in vitro* and F19 works better *in vivo*. In a murine MRSA bacteremia/sepsis model, F19 treatment alone resulted in 100% survival while untreated animals had 70% mortality. Furthermore, F19 enhances antibiotic efficacy *in vivo*. Notably, in a murine MRSA wound infection model, combination of F19 with antibiotics resulted in bacterial load reduction. Thus, F19 could be used alone or in combination with antibiotics to prevent and treat infections of Gram-positive pathogens.

## Introduction

The declining pipeline of effective antimicrobial treatments poses a worldwide threat to public health. Without drugs to treat bacterial infections common skin and respiratory infections might become deadly. Even routine procedures, such as insertion of catheters, may become life threatening without antimicrobial drugs. According to a recent report, ten million people are predicted to die of infectious diseases in the year 2050 unless action is taken to bring new treatments into the clinic^1^. Antivirulence agents represent an attractive alternative to antibiotics in monotherapy or as an adjuvant in conventional antibiotic therapy^2^. Unlike antibiotics, these agents do not kill the pathogen, and therefore there is diminished survival pressure on the pathogen to develop resistance. Instead, antivirulents disarm the pathogen of disease-causing toxins and other virulence factors that compromise the host defense response. Without the toxin-induced damage to cells, an intact host immune system is able to clear the infection. Therefore, in relatively healthy patients antivirulence therapy alone might be sufficient to combat an infection. For immunocompromised patients, a combination therapy of an antivirulent with an antibiotic might be in order. In this scenario, even an “obsolete” antibiotic to which the pathogen is resistant in monotherapy could be used because antivirulence agents have been shown to potentiate the efficacy of such obsolete antibiotics^3,4^.

**Figure 1:**
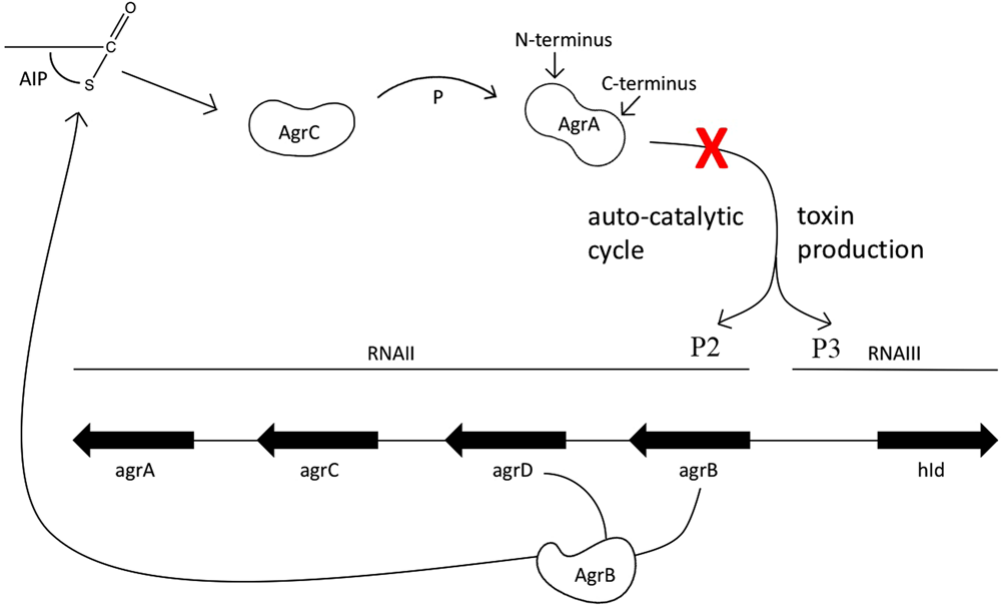
*S*. *aureus* agr operon for toxin production. The cyclic autoinducing peptide (AIP) is the signaling molecule coded for by *agrD* and processed by agrB. Mature AIP is secreted to the cell surface where it binds to and activates the histidine kinase AgrC on the same cell or on a different cell. Consequently, AgrC autophosphorylates and transfers its phosphoryl group to Asp 59 on the N-terminal domain of the response regulator protein AgrA. Phosphorylated AgrA undergoes a conformational change to form a dimer, which enables its C-terminal DNA-binding domains to bind to promoter P2 to activate AIP transcription in an autocatalytic fashion. When the AIP concentration reaches a certain threshold AgrA also binds to the tenfold weaker promoter P3, which drives the transcription of RNAIII, a master regulator of expression a series of toxins and virulence factors in the post-exponential growth phase. RNAIII encodes the hemolysin δ toxin (hld). The drug discovery target in this work is the inhibition of AgrA binding to promoter P3, as indicated by the red X.

Toxin production in S. *aureus* is predominantly controlled by the *agr* quorum sensing system (Fig. 1)^5,6^. The *agr* operon contains two promoters P2 and P3. Activation of P2 triggers the biosynthesis of the autoinducing peptide (AIP) in pro form via *agrD* and processing by the membrane-bound protease *ag*r*B*. AIP is secreted in cyclized form where it binds to and activates the membrane-bound histidine kinase AgrC, which in turn transfers a phosphate group to the response regulator AgrA. Upon phosphorylation AgrA acts as transcription factor that initially binds to promoter P2 to create more AIP, AgrC and AgrA in an autocatalytic fashion. Once a threshold level of activated AgrA is reached it binds to the weaker promoter P3, which triggers the expression of a series of toxins and virulence factors, including α-hemolysin (Hla) and phenol-soluble modulin-α (Psm-α)^7^.

**Figure 2:**
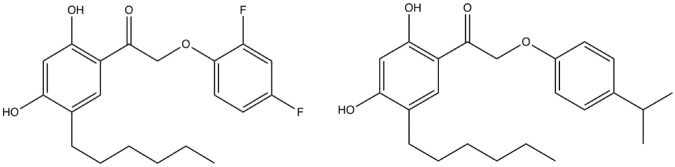
Structural formulae of biaryl hydroxyketones F12 (left) and F19 (right).

The target of this drug discovery project is the C-terminal DNA-binding domain of AgrA. In our previous studies hit compounds were identified that target AgrA and inhibit Hla transcription^8^. Upon synthesis of a combinatorial library more potent compounds were identified, including biaryl hydroxyketones F12 and F19 (Fig. 2)^9,10^. F12 is most efficacious *in vitro* whereas F19 works better *in vivo*.

Homologs of AgrA exist in many Gram-positive organisms, as shown in Supplemental Figure S1. This was the rationale for exploring F12 and F19 efficacy in Gram-positive pathogens other than S. *aureus*.

Here we describe the antivirulent properties of biaryl hydroxyketone small molecules F12 and F19 against drug-resistant Gram-positive pathogens. F12 and F19 inhibit toxin formation and exhibit *in-vivo* efficacy in animal models of MRSA wound infections and bacteremia. Of particular importance is the rescue of mice from an otherwise lethal dose of MRSA USA300 by F19 alone. These results open the prospect of successfully treating bacterial infections with an antivirulence agent without resorting to antibiotics.

## Results

### F19 binds to response regulator AgrA

**Figure 3:**
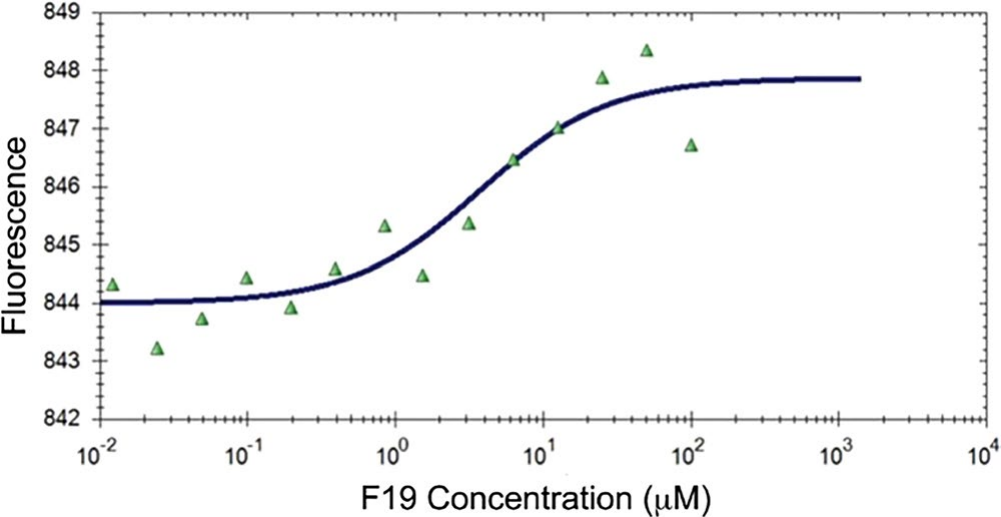
Binding curve of F19 to the C-terminal domain of AgrA from *S*. *aureus* as determined by microscale thermophoresis.

F19 and F12 bind to the C-terminal DNA-binding domain (AgrA_C) of the *Staphylococcus aureus* response regulator AgrA (residues 143–238) with affinities of 2.9 ± 0.4 and 4.5 ± 0.4 µM, as determined by microscale thermophoresis (Fig. 3). F19 also binds to AgrA_C from *Staphylococcus epidermidis* with an affinity of 2.7 ± 0.7 µM. We chose to perform these affinity measurements on the C-terminal domain since the full-length AgrA protein tends to aggregate and is difficult to work with.

### Mapping the F19-binding site on AgrA_C

**Figure 4:**
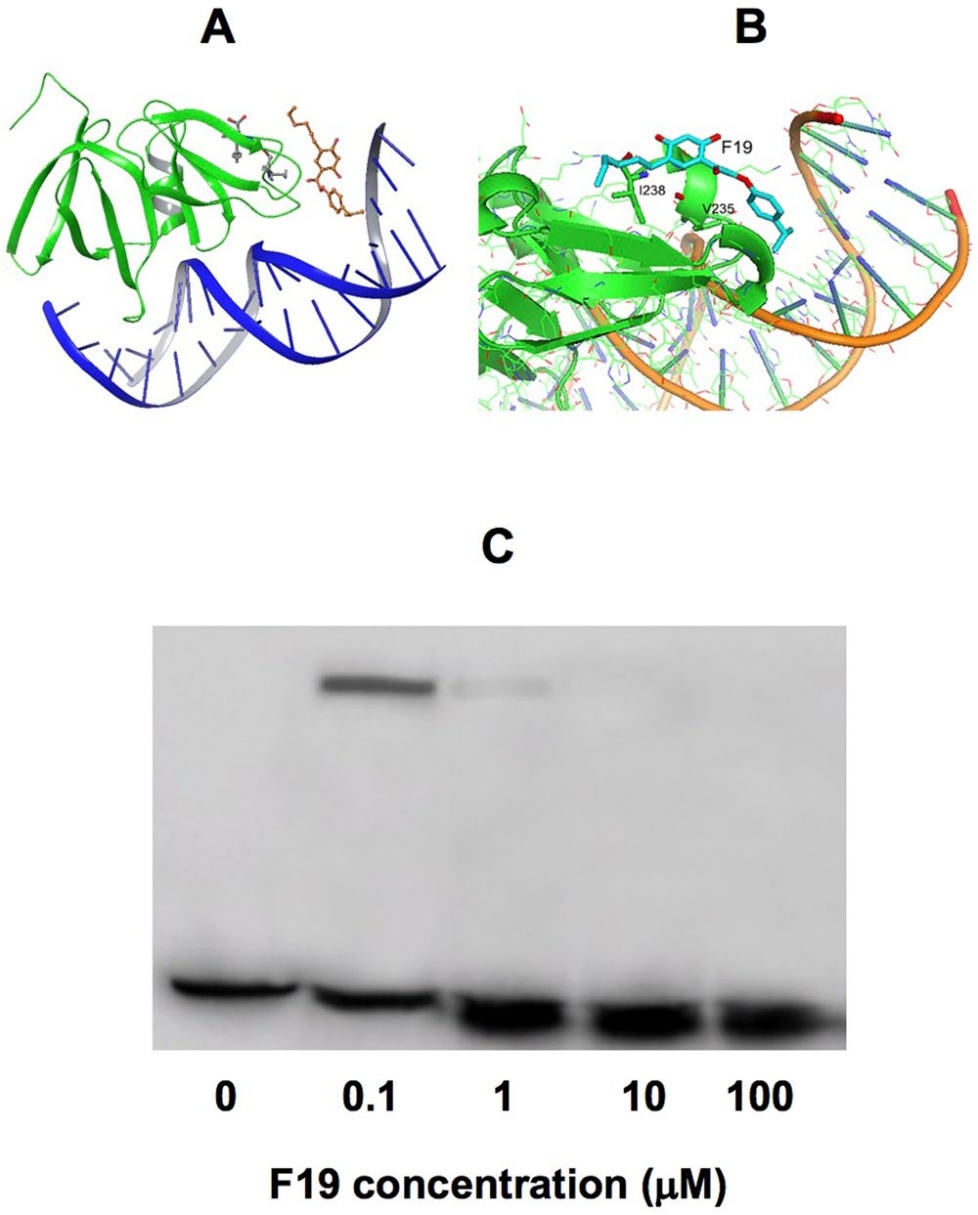
(**A**) F19 docked onto the cocrystal structure of the C-terminal domain of AgrA (AgrA_C) and a cognate oligonucleotide (PDB code 3BS1^12^). The docking was centered on the midpoint between V235 and I238 (shown in ball-and stick), two residues implicated in F19 binding by site-directed alanine mutagenesis. (**B**) Close up of the F19 binding site on the interface between AgrA_C and the DNA. (**C**) Electrophoretic mobility shift assay of AgrA_C from *S*. *epidermidis* as a function of F19 concentration. P3 DNA is an oligonucleotide corresponding to the P3 promoter sequence TAGAAACAATCTTATTTTTTTTGAATATAC. P3 DNA was radiolabeled with ^32^P. The concentration of P3 DNA was 1 nM. 1 µM AgrA_C was added in lanes 2–5. F19 was titrated in at increasing concentrations while maintaining a constant concentration of 1% DMSO. The AgrA_C-DNA complex band is present at 0.1 µM F19, can barely be seen at 1 µM F19 and is absent at 10 and 100 µM F19. This gel is in compliance with the digital image and integrity policies.

In the absence of a cocrystal structure of F19 with AgrA we attempted to map the binding site by site-specific mutagenesis. The C-terminal amino acid sequence SVRNVKKI (residues 231–238) of AgrA has been reported as a locus for small molecule interaction to inhibit DNA binding^11^. Since F19 is lipophilic, we chose to examine the involvement of the hydrophobic residues V232, V235 and I238 within the putative binding site by alanine mutagenesis of these residues. The V232A mutant exhibited wt affinity to F19, whereas the affinity was reduced 5- and 14-fold, by the V235A and I238A mutants, respectively. This result suggests the involvement of these residues in F19 binding. In order to further shed light on the mechanism of action of F19, we docked the structure of F19 onto the crystal structure of AgrA_C in complex with a cognate oligonucleotide (PDB code 3BS1)^12^. The docking was centered on the midpoint between V235 and I238, the two residues implicated in F19 binding by site-directed alanine mutagenesis (Fig. 4A,B). The location of docked F19 at the interface between AgrA and DNA is consistent with the notion of F19 impeding the association of AgrA with its cognate DNA promoter P3. This hypothesis was confirmed by an electrophoretic mobility shift assay. F19 prevented the formation of the protein–nucleic acid complex in a concentration-dependent manner (Fig. 4C).

### Stability of F12 and F19 in mouse hepatic microsomes

After incubation at 37 °C in mouse hepatic microsomes F19 was very metabolically stable, returning a half-life above the top limit of the assay format used (>100 minutes). F12 was rapidly metabolized in the assay with a half-life of 20 minutes.

### Inhibition of toxin and virulence factor transcription in MRSA

**Figure 5:**
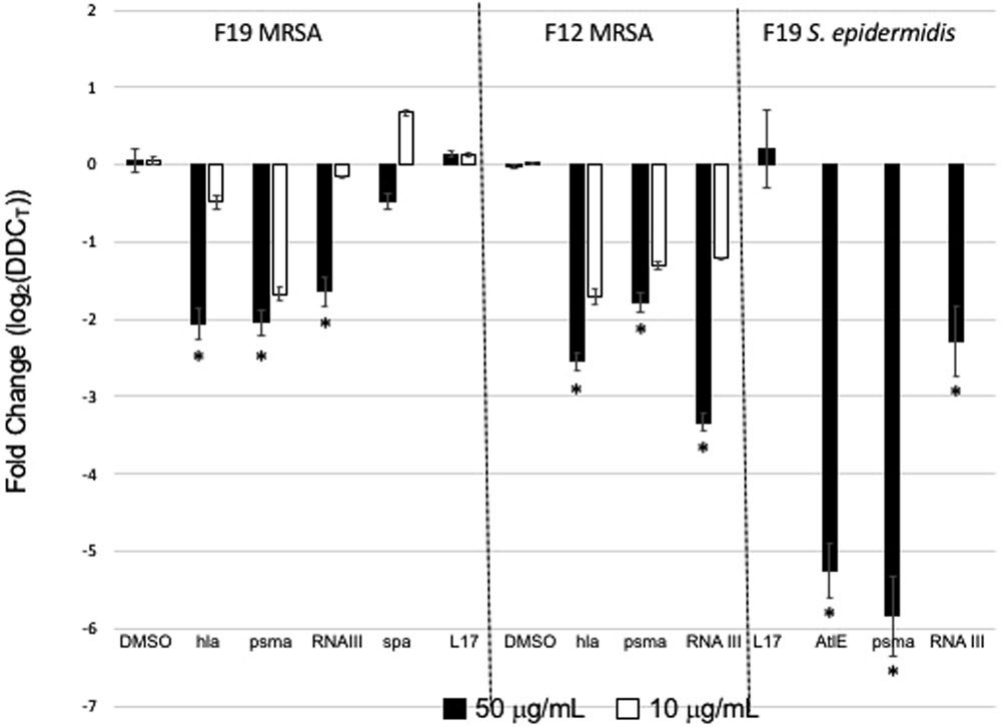
Levels of transcription of hla, psm-α, RNAIII, spa, and L17 in MRSA strain USA300 cultured in the presence of F19 (left panel) or F12 (center panel). Transcription levels of Autolysin E (AtlE) and psm-α (psma) in Methicillin-Resistant *Staphylococcu*s *epidermidis* (MRSE) clinical isolate Q-15 in the presence of F19 are shown in the rightmost panel. Compound concentrations are 50 mg/L (filled bars) or 10 mg/L (open bars). Values are averages for three separate experiments; error bars indicate standard deviations; *P < 0.05. Gene expression is displayed as log_2_ of relative gene expression with the ribosomal protein L17 used as reference.

Blocking AgrA binding to promoter P3 inhibits toxin and virulence factor transcription. This was determined by qPCR experiments of gene expression of the α-toxin (hla), the staphylococcal toxin most damaging to the host cells, phenol-soluble modulin-α (psm-α) and RNAIII, a master regulator of virulence factor production. RT-qPCR experiments were performed on RNA isolated from MRSA USA300 cultured with F19. As shown in Fig. 5A the expression of hla and psm-α was reduced in the presence of 50 mg/L F19 compared to a housekeeping gene encoding the 50S ribosomal protein L17. Expression of RNAIII, which encodes δ-hemolysin, was only moderately reduced. Expression of surface protein A (*spa*), which is implicated in proteolysis of MRSA biofilm, was not much affected. The observed effects were dose dependent since a more moderate decrease was observed in the presence of a lower concentration (10 mg/L) of F19. The downregulation in MRSA virulence factor gene transcription was not due to bactericidal or bacteriostatic effects as demonstrated by identical cell densities measured at OD_600_ or by CFU counts between the F19 treated vs. untreated cultures. Thus, we conclude that F19 is an inhibitor of quorum sensing and not a bacteriostatic or bactericidal agent.

### Inhibition of virulence factor transcription in *Staphylococcus epidermidis*

Inhibition of virulence gene transcription by F19 was also examined in Methicillin Resistant *Staphylococcus epidermidis* (MRSE). This pathogen is less virulent than MRSA as it does not contain the *hla* gene. However, *S*. *epidermidis* is notorious for adhering to surfaces and obstructing catheters, due to expressed AtlE bifunctional autolysin/adhesion protein^13^. As shown in Fig. 5B, F19 at a concentration of 50 mg/L inhibits AtlE and psm-α transcription in MRSE Q-15 cells. This result suggests broad-spectrum efficacy of F19 against *Staphylococci*.

### Inhibition of host cell lysis caused by several Gram-positive pathogens

Gram-positive pathogens compromise host cell defense factors by puncturing membranes of immune system cells^14^. To examine F19 efficacy in preventing host cell damage by various pathogens, cell lysis assays were carried out by measuring leakage of lactate dehydrogenase (LDH) from cells^15^. F19 was found to inhibit pathogen-mediated lysis of human THP-1 monocytes and mouse macrophage cell line J7774.2 by a series of Gram-positive pathogens, including MRSA, MRSE, *Streptococcus pyogenes*, *Streptococcus pneumoniae* and *Bacillus* *cereus* in a dose dependent manner (Table 1). These results indicate broad-spectrum efficacy of F19 against Gram-positive pathogens *in vitro*.

**Table 1:** Inhibition of pathogen-mediated cell damage to human THP-1 cells by F19 and F12.

% Damage (LDH Leakage)						
	(µg/mL)	MRSA USA300	MRSE Q-15	*Streptococcus pyogenes* AC7	*Streptococcus pneumoniae NR13395*	*Bacillus cereus*
Control	0	100.0 ± 1.0	100.0 ± 1.7	100.0 ± 4.0	106.7 ± 5.3	100.0 ± 3.3
F19	1	62.0 ± 0.6	85.5 ± 2.6	—	95.3 ± 1.1	—
	5	—	62.6 ± 1.1	97.4 ± 0.7	72.9 ± 1.1	—
	10	44.2 ± 2.0	48.1 ± 1.1	88.4 ± 1.9	48.6 ± 1.8	—
	20	—	26.5 ± 0.5	73.6 ± 0.5	39.6 ± 0.6	—
	30	—	22.1 ± 0.6	56.2 ± 3.4	—	63.7 ± 1.3
	40	—	19.6 ± 1.1	38.7 ± 1.8	—	52.0 ± 0.1
	50	29.1 ± 1.6	—	—	40.2 ± 0.8	69.7 ± 1.2
	0	—	100.0 ± 1.2			
	1	—	85.1 ± 1.0			
	5	—	71.9 ± 3.2			
	10	—	35.4 ± 0.5			
F12	20	—	24.3 ± 0.5			
	30	—	22.4 ± 0.4			
	40	—	19.2 ± 1.1			
	50	20 ± 3.5	—			

### ***In vivo***: F19 potentiation of β-lactam antibiotics in a MRSA wound model

Animal experiments were carried with compound F19 since preliminary experiments showed that it is more efficacious than F12.

The wound healing capacity of F19 in MRSA-infected wounds was described by us previously^9^. However, the bacterial load on the wounds had been equal to those of the non-treated controls, as is to be expected from an antivirulence agent that does not kill the pathogen. This raises the concern that the infection might recur once the treatment is stopped. To address this concern the same experiment was repeated by combination therapy of F19 with cephalothin. This antibiotic was selected because MRSA is resistant to it in mono therapy.

Wounds created on the back of mice were inoculated with 10 µl of 1 × 10^7^ CFUs of MRSA USA300. Infected mice were randomized into the following groups (5 per group); Group 1: Infected, treated with 30 mg/kg cephalothin, Group 2: infected, treated with 20 mg/kg F19, Group 3: infected, treated with 30 mg/kg cephalothin and 20 mg/kg F19, Group 4: infected, treated with vehicle control, Group 5: infected, treated with 30 mg/kg vancomycin as a positive control and Group 6: infected, untreated control. Beginning 1 h post inoculation, the treatments were administered topically twice a day for seven days. Mice were sacrificed on day 8 and the bacterial tissue burden in CFUs per gram of tissue was measured.

**Figure 6:**
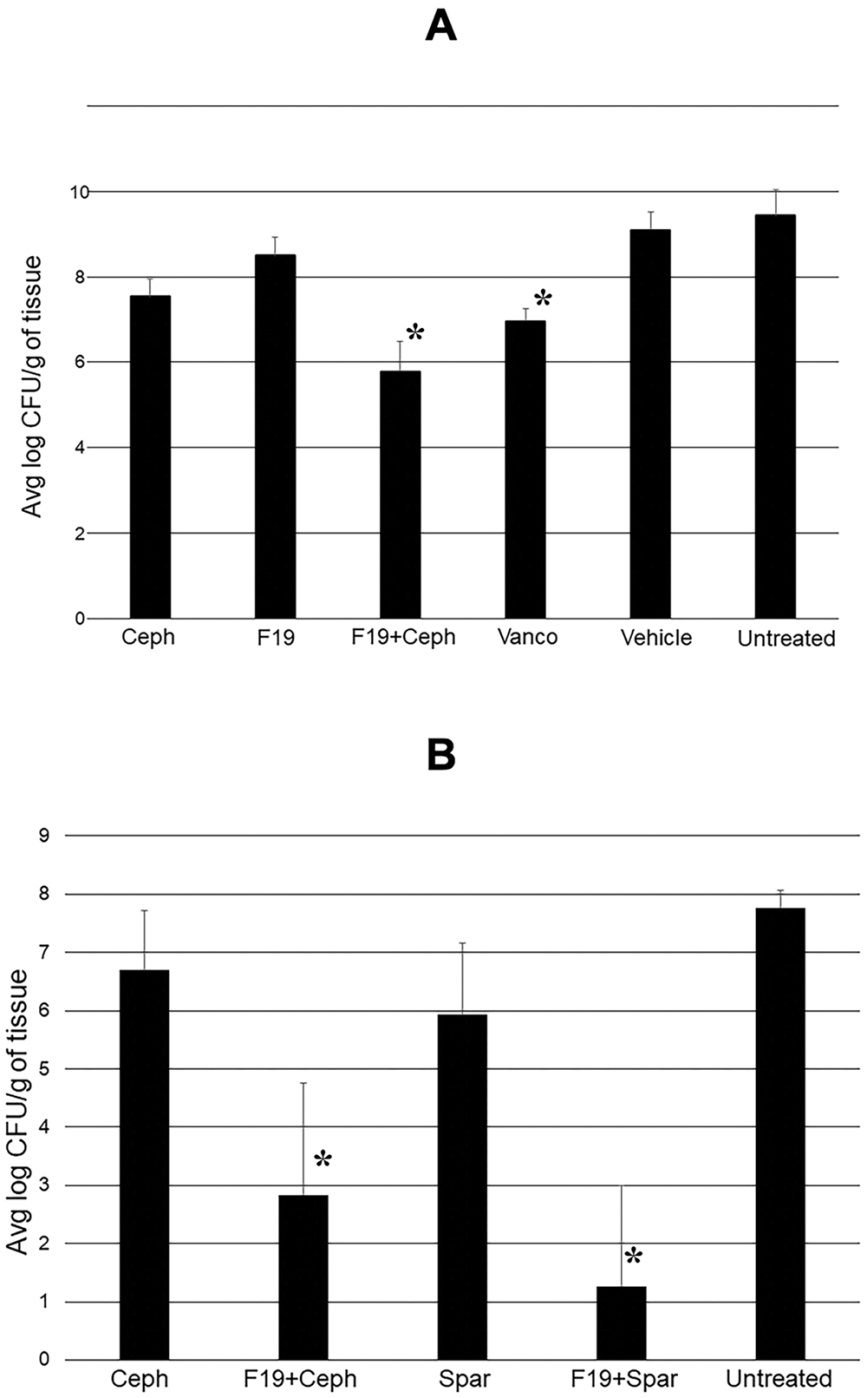
(**A**) Bacterial load on MRSA USA300 – infected wounds in mice on day 8 post inoculation. F19 was applied at 20 mg/kg, Ceph, 30 mg/kg cephalothin; Vanco, 30 mg/kg vancomycin. Treatments were applied topically twice a day. Combination therapy with 20 mg/kg F19 and 30 mg/kg cephalothin was more effective in reducing bacterial load compared to all other treatments, including vancomycin, the standard treatment of care for MRSA infections. Statistical significance is indicated by *P < 0.05. (**B**) Repeat murine wound infection experiment to probe the effect of sparfloxacin (Spar). Bacterial load on MRSA USA300 – infected wounds in mice was measured on day 8 post inoculation. Treatments were applied topically twice a day. Combination therapy with 20 mg/kg F19 and 30 mg/kg cephalothin or 30 mg/kg sparfloxacin was more effective in reducing bacterial load than mono therapy. The average log CFU/g values are for untreated 7.76 ± 0.31, 30 mg/kg cephalothin 6.69 ± .03, 30 mg/kg cephalothin +20 mg/kg F19 2.84 ± 1.92, 30 mg/kg sparfloxacin 5.93 ± 1.22, 30 mg/kg sparfloxacin +20 mg/kg F19 1.26 ± 1.73. Statistical significance is indicated by *P < 0.05.

As shown in Fig. 6A, the microbial burdens were analyzed and given as average log CFUs ± the standard deviation. As expected, the untreated and vehicle control groups had the highest bacterial burden, (average log CFUs, 9.4 ± 0.6 and 9.1 ± 0.4, respectively). The bacterial burdens for cephalothin alone and F19-alone treated groups were, 7.5 ± 0.4, and 8.5 ± 0.4, respectively on a log scale. Combination treatment with cephalothin and F19 showed a reduced tissue burden of 5.8 ± 0.7, while treatment with the vancomycin control demonstrated a tissue burden of 7.0 ± 0.3, all on a log scale. To summarize, treatment with cephalothin alone, cephalothin in combination with F19, and vancomycin resulted in significantly lower microbial burdens when compared to the untreated and vehicle controls (*P*-values of <0.05). Importantly, treatment with cephalothin in combination with F19 resulted in a significantly lower microbial burden compared to all other treatment groups (*P*-values of <0.05), including vancomycin, the standard of care for MRSA infections.

### *In vivo*: Potentiation of F19 with a fluoroquinolone antibiotic

To find out whether the potentiation of antibiotics can be extended beyond β-lactam antibiotics the same experiment was repeated in the presence of sparfloxacin, a fluoroquinolone antibiotic. A shown in Fig. 6B, combination therapy with 20 mg/kg F19 and 30 mg/kg sparfloxacin reduced the bacterial load on the wounds by about five orders of magnitude, from 5.93 ± 1.2 to 1.26 ± *1*.7 CFU on a log_10_ scale compared to mono therapy with 30 mg/kg sparfloxacin *(P*-value < 0.05). Hence, the potentiation of antibiotic efficacy appears to be independent of the antibiotic class used.

### *In vivo*: Efficacy of F19 alone and in combination in an MRSA bacteremia/sepsis model

To further study the efficacy of F19 against a systemic MRSA infection, a bacteremia/sepsis mouse model study was carried out. In this work a total of 60 female CD-1 animals divided into 6 groups of 10 mice each were used. Mice in all the groups received one intravenous injection of MRSA USA300 at a lethal dose of 1.6 × 10^10^ CFUs/mL in a volume of 100 µL into the tail vein. At 2 h post inoculation, the mice were treated intraperitoneally with 6 different agents. The treatment was repeated twice daily for seven days. Group 1 mice did not receive any treatment, group 2 mice received only vehicle injections, group 3 mice were treated with 30 mg/kg cephalothin, group 4 mice were treated with 30 mg/kg of F19, group 5 mice were treated with a combination of 30 mg/kg F19 and 30 mg/kg cephalothin, and group 6 mice were treated with 30 mg/kg vancomycin.

All animals were observed three times daily for clinical phenotype and they were scored for their health status on a scale from 1 to 7 where 1 is “completely healthy” and 7 is “dead” (Supplemental Table S2). Blood samples were collected at 24 h, 48 h, day 5 and day 7 following bacterial inoculation. All mice were euthanized after day 7.

**Figure 7:**
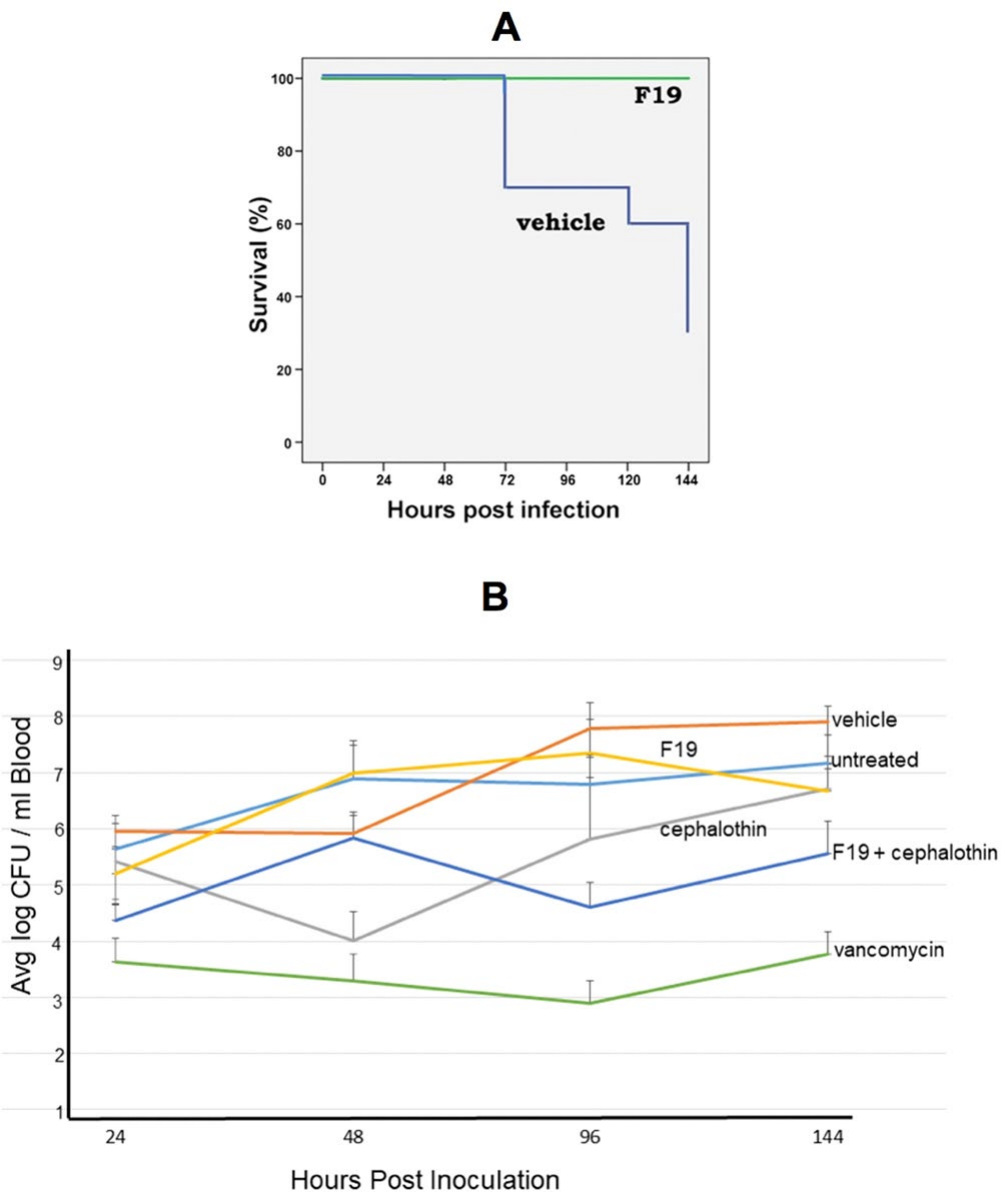
(**A**) Survival of mice inoculated with a lethal dose of 1.6 × 10^9^ CFUs MRSA USA300 and treated twice daily via IP for 7 days with 30 mg/kg F19 or vehicle. All 10 F19-treated mice survived whereas 7 out of 10 vehicle-treated mice died. (**B**) Mean bacterial load in the blood of mice as a function of time in the bacteremia/sepsis experiment. At the conclusion of the experiment the bacterial load in the blood of animals was reduced by about two orders of magnitude by combination treatment of F19 with cephalothin. Vancomycin treatment was most effective in reducing bacterial load by about 4 orders of magnitude blow vehicle control.

As shown in Fig. 7A, all 10 mice treated with F19 alone survived, whereas 7 out of 10 vehicle-treated mice died. Moreover, all ten F19-treated animals received a health score of 2, which is defined as “active, scurrying, burrowing and slightly ruffled”. 1 out of 10 cephalothin-treated animals was found dead on day 3; 6 animals received a health score of 3 and 3 animals received a score of 4. All 10 animals treated with the combination of F19 and cephalothin survived with a health score of 2, just as the animals treated with F19 alone. Mean bacterial load in F19 alone treated animals on day 6 was nearly an order of magnitude lower compared to vehicle-treated animals. Animals treated with cephalothin had approximately the same bacterial burden levels as F19-treated animals. However, treatment with a combination of F19 and cephalothin resulted in a bacterial load nearly 10-fold lower than treatment with F19 or cephalothin alone. Thus, the addition of cephalothin to F19 treatment had no impact on the health status of the animals but caused a reduction in bacterial load. Treatment with vancomycin showed the most efficacy with a bacterial load almost two orders of magnitude lower than the F19-cephalothin treatment and four orders of magnitude lower than vehicle treatment (Fig. 7B). Vancomycin-treated animals all survived and were deemed completely healthy with a health score of 1. Although F19 treatment in this experiment is somewhat inferior to vancomycin it represents a novel non-antibiotic therapy that conferred 100% survival to animals inoculated with a lethal dose of MRSA.

### Antivirulent F19 is a promising alternative treatment

Taken together, F19 represents an alternative treatment for staphylococcal infections. In the wound infection experiment, combination treatment of F19 with cephalothin, a cephalosporin antibiotic that has no efficacy against MRSA on its own, is more efficacious in reducing bacterial load than treatment with vancomycin, the current standard of care for MRSA infections. However, in the murine bacteremia/sepsis experiment, the F19-cephalothin combination is less efficacious in reducing bacterial load than vancomycin treatment. Nonetheless, mono therapy with F19 rescued all ten animals from death, and they appeared relatively healthy. This result indicates the potential of F19 to become an important weapon in the medicinal arsenal against staphylococcal infections and perhaps all infections by Gram-positive pathogens.

## Discussion

Biaryl hydroxyketone compounds F12 and F19 exhibit properties beneficial to the prevention and treatment of bacterial infections by inhibiting the formation of disease-causing toxins and potentiating antibiotic efficacy *in vivo*. The enhancement of antibiotic efficacy could be explained by a concerted action with the immune response, which remains uncompromised due to the absence of toxins.

### F12 and F19 are broad-spectrum antimicrobial agents against Gram-positive pathogens

Gram-positive bacteria all have *agr* operons that contain homologs of the *S*. *aureus* response regulator AgrA, which has been the target of this drug discovery endeavor^8,9^. Therefore, it is not surprising that an inhibitor of AgrA in *S*. *aureus* would have similar activity in other Gram-positive organisms. This explains the efficacy of F12 and F19 against *Staphylococci*, *Streptococci* and *Bacilli*, including drug-resistant strains that pose a threat in public and clinical health care. Efficacy of F12 and F19 against these pathogens is demonstrated here *in vitro* by downregulation of toxin expression and inhibition of host cell lysis.

Amongst the sixteen two-component regulatory systems (TCRSs) in *S*. *aureus*, *agr is the most important* TCRS for toxin and virulence factor production^16–18^. In principle, any component of the *agr* operon could have been targeted in order to inhibit toxin production^5^. AgrB and AgrD are membrane proteins involved in the expression, maturation and secretion of the autoinducing peptide (AIP) that functions as the signaling molecule in Gram-positive bacteria. AgrC is a large membrane-bound histidine kinase that gets activated by the AIP. There are four sequence variants of the AIP and four corresponding variants of AgrC. By contrast, AgrA is a unique small and soluble protein, making it the most suitable drug target within the *agr* operon. Other reported small-molecule inhibitors of AgrA include savirin^19^, hydroxyemodin^20^ and phloretin^21^. Additionally, several natural products have been reported to inhibit the *agr* system, although it is not clear which component of *agr* is inhibited in these studies^22–24^. *Agr*-targeting quorum quenchers with demonstrated *in-vivo* activity have recently been reviewed by Salam and Quave^25^.

### F12 and F19 are distinct among small molecule antivirulence agents

F12 and F19 are distinct from other *agr* inhibitors in that they are novel synthetic molecules designed from first principles based on the crystal structure of AgrA. Importantly, here we demonstrate for the first time that an antivirulence agent can cure bacteremia in an animal model without resorting to an antibiotic.

F19 is also efficacious as adjuvant in conventional antibiotic therapy in animal models, even with antibiotics to which MRSA and MRSE are resistant in mono therapy. This raises the interesting prospect of bringing back “old” legacy antibiotics, such as penicillin, for use in the clinic in combination with F19, in much the same way as combination therapy of a β-lactam antibiotic with a β-lactamase inhibitor, such as amoxicillin and clavulanic acid, has been used clinically for years. Other adjuvants to antibiotic therapy have been described in the literature but have not been introduced into the clinic^26–29^. These adjuvants are mostly natural compounds that seem to enhance antibiotic efficacy by facilitating penetration of the antibiotic into the bacterial cell.

### Combination therapy of antibiotics with an antivirulence agent

Combination therapy of an antibiotic with an antivirulence agent is a new concept that has yet to get regulatory approval^30^. Legacy antibiotics are off patent and affordable in generic form, thereby reducing health care costs. However, the cost of antivirulence agents could offset the savings. Nonetheless, antibiotic-antivirulent combination therapy constitutes a potential new mode of therapy that may alleviate the looming antibiotic resistance crisis.

### Efficacy shown *in vivo*

F19 is more efficacious *in vivo* than F12, apparently due its enhanced metabolic stability, as determined in mouse liver microsomes. Thus, it seems that F19 is a poorer substrate for P450 liver enzymes than F12.

*In vivo* efficacy has been established here in murine models of MRSA wound infections and bacteremia/sepsis. While mono therapy with F19 in MRSA-infected wounds did not reduce the bacterial load on the wounds, combination therapy of F19 with cephalothin, a β-lactam antibiotic to which MRSA is resistant, lowered the bacterial load by about three orders of magnitude compared to vehicle-treated or untreated wounds. This combination treatment was more efficacious than vancomycin, the standard of care for MRSA infections, by about one order of magnitude.

In the murine MRSA bacteremia and sepsis model treatment with F19 alone rescued all 10 animals from death while 7 out of 10 vehicle-treated mice died. Moreover, all F19-treated mice were deemed relatively healthy at the end of the 7-day treatment period. In this experiment vancomycin was superior to F19 alone or in combination with cephalothin in terms of bacterial load and health status. However, this experiment indicates that treatment with F19 alone results in 100% survival, in accordance with the well-established role of the *agr* system in *S*. *aureus* bacteremia^31^. F19 treatment caused only a 10-fold reduction in bacterial load on day 7. The question is why was the host immune system not able to completely clear bacteria that were compromised in their virulence? The answer likely lies in the requirement of the immune response to be primed by the toxins. Since F19 inhibits toxin formation there was likely not enough toxin around to activate efficient clearing by the immune system.

### Implications for clinical research

This result raises the question of whether bacterial infections could be cured without antibiotics. The answer likely depends on the overall health status of the patient. In a relatively healthy individual suffering from a community-associated MRSA infection, therapy with an antivirulence agent, such as F19, might be enough to cure the infection. Antivirulence therapy would leave host defense factors intact and ready to clear the infection. However, in immunocompromised patients combination therapy with an antibiotic might be in order.

In conclusion, F19 is a promising alternative to antibiotics or an adjuvant in conventional antibiotic therapy. F19 might provide a partial answer to the looming threat of antimicrobial resistance.

## Methods

### Reagents, proteins, microorganisms and eukaryotic cells

Reagents and culture media were purchased from Fisher Scientific except for cephalothin (MP Biomedicals, Solon, OH). All solutions were made with sterile ultrapure deionized water. Buffers and culture media were sterilized by autoclaving at 121 C for 25 min.

MRSA USA300 is a clinical isolate from a patient at Metro Health Medical Center, Cleveland, OH. MRSE Q-15, *Streptococcus pyogenes and Streptococcus pneumoniae* were clinical isolates obtained from the bacteriology laboratory at University Hospitals, Cleveland, Ohio. *Bacillus anthracis* strain Ames was used at a BSL-3 facility at Southern Research, Birmingham, AL.

Human THP-1 monocytes and mouse J774.2 macrophages were obtained from Clifford Harding in the Department of Pathology at Case Western Reserve University.

### AgrA_C (MRSE) cloning, expression and purification

AgrA_C from MRSE was isolated from MRSE genomic cDNA using the primers Forward: 5′CATATGGAAAGTAATGTAGATACGATTGAGTTAAAACG3′ and Reverse: 5′AAGCTT TTATATTTTTTTAACATTACGTACTGA 3′ and cloned into TOPO TA cloning vector (Invitrogen). The fragment was then excised from the TOPO vector with NdeI and HindIII and subcloned into pET28b expression vector providing a histidine tag. *E*. *coli* strain BL21 LysS was transformed with the plasmid and used for protein expression.

Two liters of *E*. *coli* strain BL21 Lys S transformed with pET28b MRSE AgrA_C were grown shaking at 37 C to mid logarithmic phase (OD_600_ = 0.8). IPTG was added to a final concentration of 500 µM to induce protein expression. The bacteria were further cultured for 4–6 h and then harvested. Bacteria were pelleted, frozen and resuspended in 25 mM HEPES pH7.2, 1 M NaCl, 25% glycerol and sonicated with a Branson 450 sonifier 4 times for 1 min to lyse the cells. The cells were centrifuged and the supernatant was loaded onto a Talon (Cobalt) affinity column. Bound protein was washed and eluted with a gradient of 10–500 mM imidazole containing buffer (25 mM HEPES pH7.2, 1 M NaCl, 25% glycerol) in 20, 5 mL fractions. Protein containing fractions were pooled, dialyzed and concentrated and loaded on to a Sephadex 75 increase molecular filtration column (GE Life Sciences). Fractions corresponding to the monomer form of the protein were collected and used for subsequent experiments.

### AgrA_C (MRSA) expression and purification

The plasmid containing AgrA_C from S. *aureus* was obtained from the laboratory of Pamela Hall at the University of New Mexico in Albuquerque, NM. This plasmid contained the C199S mutation and was subcloned into a pET28a vector (Novagen) containing a His tag for purification^32^. This plasmid was transformed into BL21 DE3 cells. Protein expression and purification was performed as described above for AgrA_C from MRSE except that the overnight incubation was carried out at 18 C.

### Microscale Thermophoresis (MST)

The AgrA_C proteins from *S*. *aureus* and *S*. *epidermidis* were fluorescently labeled with Alexa Fluor 647 NHS ester and purified on Zeba spin columns. Compound F19 was diluted as a gradient through the capillaries while protein concentrations were maintained constant. Measurements were performed on a Nanotemper Monolith NT.115 apparatus. Lysozyme and known inhibitor N,N’,N”-triacetylchitotriose (Fisher Scientific) were used as controls.

### Electrophoretic Mobility Shift Assay (EMSA)

An oligonucleotide corresponding to the P3 promoter sequence TAGAAACAATCTTATTTTTTTTGAATATAC from *S*. *aureus* and *S*. *epidermidis* (Integrated DNA Technologies, Inc.) was end labeled with radioactive ^32^P. The EMSA procedure was carried out as described previously^8^ except that titration with increasing concentrations of F19 was kept at a constant DMSO concentration of 1%.

### Stability Measurements of F12 and F19 in Mouse Hepatic Microsomes

A microsomal stability assay was performed at BioFocus, Ltd. (now part of Charles River, Inc.), using pooled hepatic mouse microsomes (Xenotech/1110071). F12 and F19 as well as control compounds (midazolam, dextromethorphan), were prepared in DMSO and incubated at an initial concentration of 1 μM in 0.25% DMSO with microsomes (0.25 mg protein/mL) at 37 °C in the presence of the cofactor.

NADPH (1 mM). Aliquots were removed at 0, 5, 10, 20 and 40 minutes for termination of reactions and compound extraction with acetonitrile containing an analytical internal standard. Samples were centrifuged and the supernatant fractions analyzed for parent compound by mass spectrometry (LC-MS/MS). The amount of compound remaining (expressed as %) was determined from the MS response in each sample relative to that in the t = 0 samples (normalized for internal standard). Ln plots of the % remaining were used to determine the half-life for compound disappearance using the relationship: t1/2 (min) = −0.693/λ where λ is the slope of the Ln % remaining vs time curve.

### Docking of F12 and F19 onto the crystal structure of AgrA_C

The compounds F19 and F12 were optimized for ligand-protein docking using Schrödinger™ LigPrep software. The crystal structure of the AgrA LytTR (C-terminal) domain bound to DNA (PDB code 3BS1) was used for the docking^12^. A cubic receptor grid of 20 Å centered around residues 235 and 238 was used. The Glide module of Schrödinger™ was used to dock molecules F19 and F12 with an OPLS-2005 force field.

#### RNA isolation

RNA was isolated according the methods described in ref.^8^. Total RNA from MRSA strain USA300 was isolated after 6 h of incubation (1:100 dilution of overnight culture in 20 mL LB) using Trizol reagent according to common RNA isolation protocols^8^. The RNA yield and purity were assayed by UV absorbance and the integrity of the RNA was assessed on a 1% denaturing agarose gel by visualizing the intact 23S, 16S rRNA bands.

#### Real-Time RT-PCR

Quantitative reverse transcription-PCR (qRT-PCR) was conducted on RNA isolates and the levels of hla, psm-α, RNAIII, spa, and L17 were assessed. The data were analyzed using the DDC_T_ method. Samples cultured with 2% DMSO were used as control. The gene corresponding to the DNA binding protein hup was used as reference housekeeping gene.

5 µg of total RNA was treated with RNAse-free DNAse (Roche) to eliminate DNA backgrounds. 1 µg of the DNAse-treated RNA was then used in a first strand reverse transcription reaction (M-MuLV Reverse transcription, NE Biolabs) with oligo dT and random hexamer primers. cDNA volumes equivalent to 10 ng of starting total RNA were used in the qPCR reactions. Real-time PCR was conducted using a SYBR green mixture (Roche FastStart Universal SYBR green Master 2x concentrated). Primer sequences for MRSA have been listed previously (7) except for MRSA L17 which are: forward primer AAACTACAGAAGCTCGTGCAAA, reverse primer TTAGCTGCATTACGACGAGAAG. The primers used for MRSE qPCR are for AtlE forward 5′TGTCCTGCTTTCACGTATGA3′ and reverse 5′ TCTTTGGAATTGGTGCAT TT3′; for RNAIII forward 5′ GCCGTGAGCTTGGGAGAGAC 3′ and reverse 5′ GACTCATATCACAGAGATGTGATTG 3′; for Psm-α forward 5′ ATGGCAGATGTAATCGCTAA 3′, and reverse 5′ TTATTTTTGAGTAAATTGATCAATTAAGCC 3′, MRSE L17 forward 5′ AGCATCTCGTCGTAATGCTGCT3′, reverse 5′ ACGAGTGTAACCACCTTGACGT 3′.

### Cell damage assays

Damage to host cells was measured by leakage of lactate dehydrogenase (LDH) with the CytoTox 96 Non-Radioactive Cytotoxicity LDH Assay Promega Kits G178 and G1782 according to manufacturer’s instructions^15^. Overnight cultures of the bacterial strain to be tested were spun down, washed, diluted 1:4 and incubated with broth containing 10–50 µg/mL of F19 or containing 2% DMSO as control. The bacteria were cultured at 37 C, in a shaking incubator for 6 h at which time 20 µL of bacterial culture was added to 5 × 10^4^ THP-1 monocytes or J774.2 macrophages in 100 µL of cell media (DMEM for J774.2 cells and RPMI for THP-1 cells) in 96 well plates. Bacteria and macrophages/monocytes were incubated together for 40 min at 37 C. Plates were then spun at 250 g for 4 min and 50 µL of the supernatant was removed to a new 96 well plate. 50 µL of substrate mix + assay buffer were added and incubated at ambient temperature for 10–30 min depending upon the rate of product formation and stopped by addition of 50 µL stop solution. The colorimetric products were read at 490 nm on a Victor 3 plate reader. The data presented are from experiments performed in triplicate.

### Murine MRSA wound infection model

All procedures in the protocol were in compliance with the Animal Welfare Act, the Guide for the Care and Use of Laboratory Animals, and the Office of Laboratory Animal Welfare at Case Western Reserve University. Procedures were as described previously^9^. Briefly, mice were anesthetized by administering ketamine, and xylazine, intraperitoneally. A 3 × 3 cm midline back area was delineated, shaved and depilated. The dorsal area was prepared for wounding using a Betadine scrub and wiping with 70% alcohol. A stainless-steel wire ring (16 mm diameter and 19 gauge) was secured to the skin with wound clips 2–5 mm to the left of midline. After splint placement, 6 mm full thickness excisional wounds were created with a punch biopsy tool in the center of each splint. The infected areas were inoculated with 10 µl of 1 × 10^7^ CFUs of MRSA USA300. After infection of the wounds, a separate sterile wound dressing was placed over the infected area.

Infected mice were randomized into the following groups (5 per group); Infected treated with cephalothin 30 mg/kg, infected treated with F19 20 mg/kg, infected treated with cephalothin 30 mg/kg and F19 20 mg/kg, infected treated with vehicle control, infected treated with vancomycin 30 mg/kg (positive control) and infected untreated control.

Beginning 1 h post inoculation, treatments were administered topically twice a day for 7 d. Tegaderm was removed daily and wounds measured with electronic calipers along diameters parallel and perpendicular to the mouse axial skeleton. Animals were treated and the Tegaderm was replaced.

Mice were sacrificed one day after the last day of treatment (day 8), and the infected area was removed aseptically and weighed. Tissue was homogenized and serially diluted in saline. The homogenates were cultured for 48 h on BHI plates to determine the colony forming units (CFUs); tissue burden is expressed as CFUs /g of tissue.

At the end of the study, all surviving animals were sacrificed by CO_2_ asphyxiation and disposed to the animal resource center for incineration.

A statistical analysis of microbial tissue burden data was performed. Significance was determined using a T-test to evaluate wound measurements and an ANOVA with a bonferroni post hoc to evaluate tissue burden. The treated groups were compared to determine treatment efficacy.

### Murine MRSA bacteremia/sepsis model

This experiment was carried out by Noble Life Sciences, Inc. (1500 Fannie Dorsey Rd, Sykesville, MD 21784).

MRSA USA300 was grown on blood agar for broth inoculation. The assay medium used to grow the bacteria was Trypticase Soy Broth (TSB). Dehydrated media was dissolved in deionized H_2_0 and subsequently autoclaved for 15 min at 121 °C. The media was cooled before using.

#### Preparation of MRSA USA300 for inoculum

A single colony of MRSA USA300, grown on trypticase soy agar (TSA) with 5% sheep blood was used to inoculate 15 tubes containing 40 mL of TSB. The cultures were incubated overnight at 37 C. Following the incubation 6 tubes were centrifuged at 4000 rpm for 10 min, and each bacterial pellet was resuspended in 40 mL of Dulbecco’s Phosphate Buffered Saline (DPBS). The cultures were combined and the OD_600_ was determined. The concentration was adjusted to 1 × 10^10^ CFUs/mL and placed on ice for inoculation to animals.

#### Determination of bacterial load in mouse blood

Bacterial counts were determined from blood samples obtained from each mouse at 24, 48, 96 and 144 h post infection. Each sample was diluted in serial logarithmic increments such that a total of 7 dilutions were performed. 40 µL of dilutions 10^–2^, 10^–4^, and 10^−6^ were plated for all animals in groups 1–5, and dilutions 10^−1^, 10^−2^ and 10^−3^ were plated for animals of group 6. All dilutions were plated in duplicate on TSA +5 % sheep blood and incubated for 24 h at 37 C. Following an overnight incubation, the colonies were counted and CFUs/mL of blood was determined.

#### Treatments

A total of 60 female CD-1 animals were used in this study. The animals were 6–8 weeks upon receipt and 7–9 weeks old at dose initiation. 60 mice were distributed into six groups of 10 mice each. The mice in all the groups received one intravenous injection of MRSA USA300 strain at a lethal dose of 1.6 × 10^10^ CFUs in 100 µL into the tail vein. At 2 h post bacterial inoculation the mice were treated intraperitoneally with six different agents. Mice of group 1 did not receive any treatment. Group 2 mice received only vehicle injections. Group 3 mice were treated with 30 mg/kg cephalothin. Group 4 mice were treated with 30 mg/kg of F19. Group 5 mice were treated with 30 mg/kg each of F19 and cephalothin. Group 6 mice were treated 30 with mg/kg vancomycin. For all the treatments, the second dose were administered 8 h post the first dose on day 1 and at 12- h intervals on subsequent days.

All the animals were observed three times daily for any clinical phenotype and scored the health status as per Supplemental Table S2. Any animal that appeared very sick and extremely lethargic was humanely euthanized. Blood samples were collected at 24, 48, 120 and 144 h following bacterial inoculation. All the mice were euthanized after day 7.

### Animal Welfare Approval

Approval for the experiments in mice was obtained from the Animal Welfare Committee at Case Western Reserve University under IACUC number 2014-0058 in accordance with the guidelines of the National Institute of Health for the use of experimental animals.

## Electronic supplementary material

Supplementary Information
