# Supplementary material for: Small-molecule AgrA inhibitors F12 and F19 act as antivirulence agents against Gram-positive pathogens
Source: Sci Rep. 2018 Oct 1;8:14578. doi: 10.1038/s41598-018-32829-w (PMC6167350; doi:10.1038/s41598-018-32829-w)
Supplement: Supplementary file 1 — Supplementary Information [file 41598_2018_32829_MOESM1_ESM.docx]

**Small-molecule AgrA inhibitors F12 and F19 act as antivirulence agents against Gram-positive pathogens**

Michael Greenberg^1#^, David Kuo^1#^, Eckhard Jankowsky^2^, Lisa Long^3^, Chris Hager^3^, Kiran Bandi^1,4^, Danyang Ma^1^, Divya Manoharan^1^, Yaron Shoham^5^, William Harte^6^, Mahmoud A. Ghannoum^3^ and Menachem Shoham^1,5,7,*^

Running Title: Antivirulence agent F19 against Gram-positive pathogens

^1^Department of Biochemistry, Case Western Reserve University School of Medicine, Cleveland, OH 44106

^2^Center for RNA and Therapeutics, Case Western Reserve University, Cleveland, OH 44106

^3^Department of Dermatology, Case Western Reserve University School of Medicine, Cleveland, OH 44106

^4^Current address, University of Pittsburgh School of Medicine

^5^Q2 Pharma, Ltd.

^6^Case Western Reserve University, Cleveland, OH 44106

^7^Lead Contact

# These authors contributed equally

*Correspondence: menachem.shoham@case.edu

**Supplemental Table S1: Health status of animals in murine bacteremia model**

| **Score** | **Initials** | **Description** | **Appearance** | **Mobility** | **Attitude** |
| --- | --- | --- | --- | --- | --- |
| 1 | H | Healthy | Smooth coat, bright eyes | Active, scurrying, burrowing | Alert |
| 2 | SR | Slightly ruffled | Slightly ruffled coat (usually only around head and neck) | Active, scurrying, burrowing | Alert |
| 3 | R | Ruffled | Ruffled coat throughout body. A "wet" appearance. | Active, scurrying, burrowing | Alert |
| 4 | S | Sick | Very ruffled coat. Slightly closed, inset eyes | Walking, but no scurrying. | Mildly  lethargic |
| 5 | Vs | Very sick, recommend euthanasia | Very ruffled coat, closed, inset eyes | Slow to no movement, will return to upright position if put on its side. | Extremely lethargic |
| 6 | E | Euthanize | Very ruffled coat, closed, inset eyes. Moribund, requiring humane euthanasia | No movement or uncontrollable spastic movements. Will not return to upright position if put on its side. | Completely  unaware or in noticeable distress |


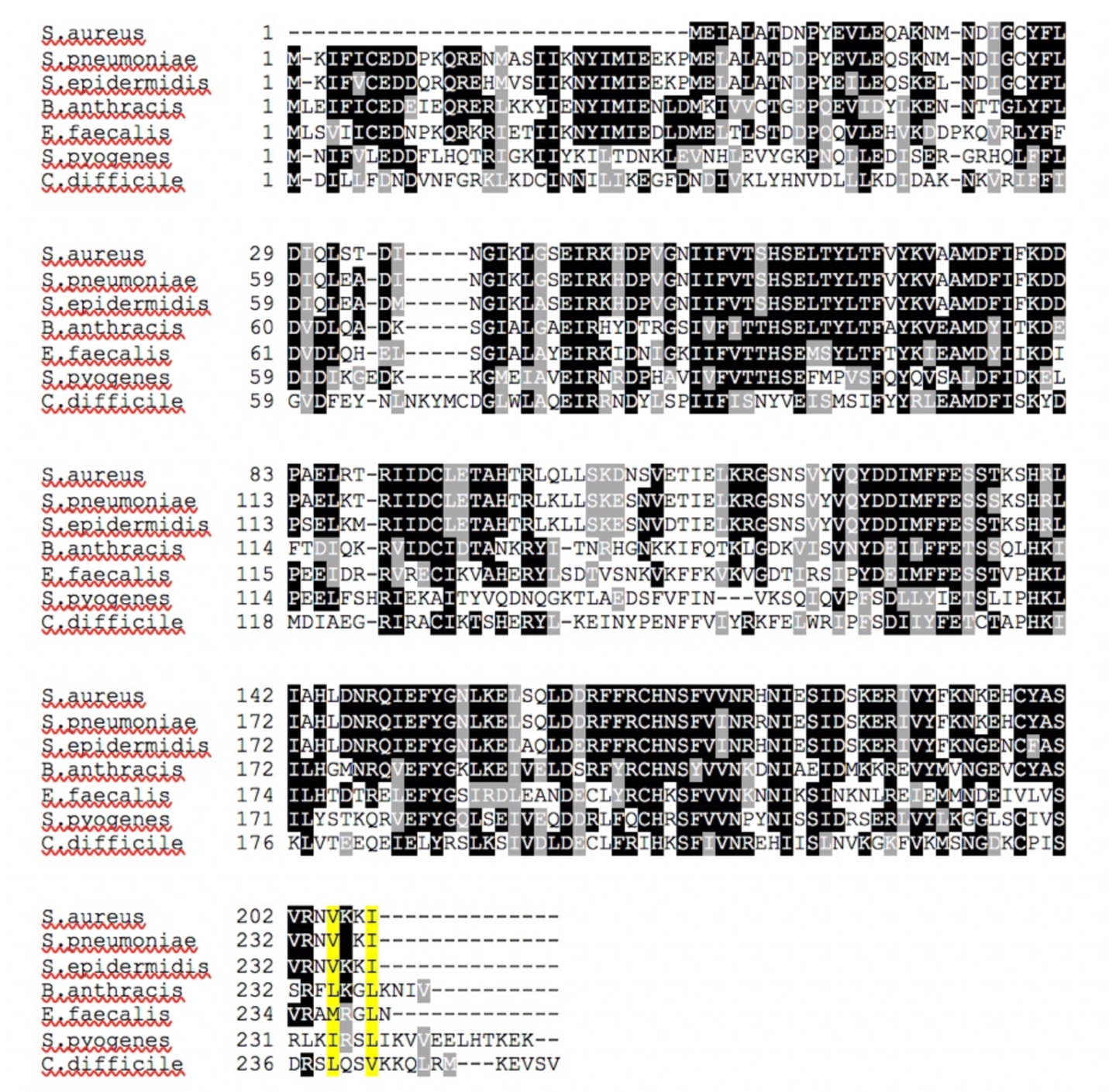


**Percentage of Sequence Identity**

S. *aureus* 100.0

S. *pneumoniae* 93.8

S. *epidermidis* 88.0

B. *anthracis* 49.8

E. *faecalis* 45.2

S. *pyogenes* 35.0

C. *difficile* 29.0

**Supplemental Figure S1: Amino acid sequence alignment of AgrA homologs in Gram-positive pathogens.** Identical residues are shaded in black and similar residues in gray. Binding of F19 to AgrA is weakened by alanine mutagenesis of V235 and I238, suggesting the involvement of these two residues in binding F19. These two residues are highlighted in yellow.

S. *aureus* AgrA is GenBank: ABB17378.1; S. *epiderimidis* is DNA-binding response regulator NCBI Sequence: WP_002485617.1; Streptococcus *pneumoniae* response regulator of the LytR/AlgR family GenBank*:* COE33874.1; Streptococcus *pyogenes* is DNA-binding response regulator NCBI Sequence WP_011528343.1; Enterococcus *faecalis* is DNA-binding response regulator NCBI Sequence WP_010777489; Bacillus *anthracis* is DNA-binding response regulator NCBI Sequence PED52039.1;

Clostridium *difficile* is DNA-binding response regulator NCBI Sequence WP_054269367.1.
